# Reactive Oxygen Species Mediate 6c-Induced Mitochondrial and Lysosomal Dysfunction, Autophagic Cell Death, and DNA Damage in Hepatocellular Carcinoma

**DOI:** 10.3390/ijms222010987

**Published:** 2021-10-12

**Authors:** Senzhen Wang, Xiaojuan Xu, Delu Che, Ronghui Fan, Mengke Gao, Yue Cao, Chaochao Ge, Yongli Feng, Jinghua Li, Songqiang Xie, Chaojie Wang, Fujun Dai, Lei Gao, Yuxia Wang

**Affiliations:** 1 Key Laboratory of Natural Medicine and Immuno-Engineering, Henan University, Kaifeng 475004, China; wangsz8836@vip.henu.edu.cn (S.W.); 104753190970@henu.edu.cn (D.C.); fanronghui@henu.edu.cn (R.F.); mengke0316@henu.edu.cn (M.G.); caoyue@henu.edu.cn (Y.C.); 104752170094@vip.henu.edu.cn (C.G.); 104753180989@vip.henu.edu.cn (Y.F.); lijinghua@henu.edu.cn (J.L.); wcjsxq@henu.edu.cn (C.W.); gaolei@vip.henu.edu.cn (L.G.); 2 School of Pharmacy, Henan University, Kaifeng 475004, China; xuxiaojuahn@163.com (X.X.); xiesq@henu.edu.cn (S.X.); 3 College of Chemistry and Chemical Engineering, Henan University, Kaifeng 475004, China

**Keywords:** reactive oxygen species, autophagic cell death, lysosomes, DNA damage, mitochondria

## Abstract

Increasing the level of reactive oxygen species (ROS) in cancer cells has been suggested as a viable approach to cancer therapy. Our previous study has demonstrated that mitochondria-targeted flavone-naphthalimide-polyamine conjugate 6c elevates the level of ROS in cancer cells. However, the detailed role of ROS in 6c-treated cancer cells is not clearly stated. The biological effects and in-depth mechanisms of 6c in cancer cells need to be further investigated. In this study, we confirmed that mitochondria are the main source of 6c-induced ROS, as demonstrated by an increase in 2′,7′-dichlorodihydrofluorescein diacetate (DCFH-DA) and MitoSox fluorescence. Compound 6c-induced mitochondrial ROS caused mitochondrial dysfunction and lysosomal destabilization confirmed by absolute quantitation (iTRAQ)-based comparative proteomics. Compound 6c-induced metabolic pathway dysfunction and lysosomal destabilization was attenuated by N-acetyl-L-cysteine (NAC). iTRAQ-based comparative proteomics showed that ROS regulated the expression of 6c-mediated proteins, and treatment with 6c promoted the formation of autophagosomes depending on ROS. Compound 6c-induced DNA damage was characterized by comet assay, p53 phosphorylation, and γH2A.X, which was diminished by pretreatment with NAC. Compound 6c-induced cell death was partially reversed by 3-methyladenine (3-MA), bafilomycin (BAF) A1, and NAC, respectively. Taken together, the data obtained in our study highlighted the involvement of mitochondrial ROS in 6c-induced autophagic cell death, mitochondrial and lysosomal dysfunction, and DNA damage.

## 1. Introduction

At present, cancer is one of the most life-threatening diseases, and cell death induced by anticancer agents in targeted cancer cells is a primary therapeutic aim of most cancer therapies [1]. Three different types of cell death, including apoptosis, necrosis, and autophagic cell death (ACD), are relatively well-characterized [2].

Autophagy is a cellular stress response and a highly regulated catabolic process that involves the formation of a double-membrane bound structure termed the autophagosome, which fuses with lysosomes for the bulk degradation of long-lived proteins and recycling of damaged organelles [3]. The process of autophagy begins with autophagosome formation mediated via several autophagy-related (Atg) proteins, including ATG5, Beclin 1 (ATG6), ATG7, LC3 (ATG8), ATG10, and ATG12. Autophagy was initially identified as a survival strategy; however, impaired or prolonged activation of this self-degradation process could also lead to cell death [4,5]. ACD, also known as type II programmed cell death, is first accompanied by large-scale autophagic vacuolization in the cytoplasm and a vacuolated cellular appearance [6]. Some anticancer drugs induce cell death by triggering autophagy, indicating that ACD may be an important factor for suppressing tumors during cancer therapy [7].

The cellular response to DNA damage includes induction of the DNA repair process to increase survival or initiation of apoptosis to remove excessively damaged cells [8]. Cell death following DNA damage is a regulated process involving molecular signals to survive or die that are dependent on a variety of factors involved in autophagy, apoptosis, senescence, and necrosis, including p53, poly [ADP-ribose] polymerase 1 (PARP-1), Bcl-2 family proteins, the mammalian target of rapamycin (mTOR), c-Jun N-terminal kinases (JNK), and ROS [9].

ROS encompass H_2_O_2_, superoxide (O_2_^−^), and hydroxyl radicals generated by all aerobic organisms [10]. A lasting increase in ROS levels causes permanent oxidative stress, leading to cellular damage, apoptotic cell death, pyroptosis, and dysfunction of organ systems [11,12,13]. Accumulating evidence suggests that cancer cells have higher levels of ROS than normal cells [14]. Recently, specifically increasing the level of ROS in cancer cells has been suggested as a viable approach to cancer therapy, leading to ROS-mediated cancer cell death [15,16].

Naphthalimides have abundant anticancer properties, but dose-limiting toxicities restrict their use. Compound 6c is a novel synthetic mitochondria-targeted flavone-naphthalimide-polyamine conjugate. Recently, we reported that 6c localized in the mitochondria and selectively induced cancer cell death [17]. However, the mechanisms underlying 6c-induced cell death, ROS generation, lysosome function, and DNA damage remains unknown. Therefore, the aim of this study was to investigate the detailed mechanisms of 6c with respect to ROS, autophagy, apoptosis, and DNA damage.

## 2. Results

### 2.1. Compound 6c Induced Extensive Cytoplasmic Vacuolations

**Figure 1:**
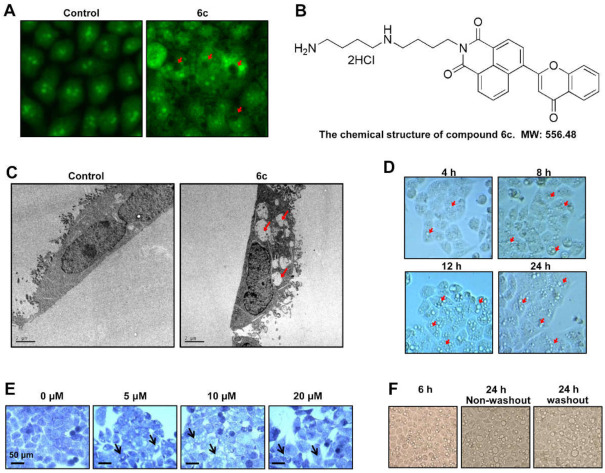
Compound 6c induced extensive cytoplasmic vacuolations. (**A**) HepG2 cells were treated with compound 6c at 10 μM for 6 h and stained with AO. Images were captured by HCS. Red arrows indicated the sites that were not stained with AO. (**B**) Chemical structure of compound 6c. (**C**) TEM images of HepG2 cells treated with 6c. (**D**) Cells were treated with 6c (10 μM) for 4, 8, 12, and 24 h, respectively. Images were captured. (**E**) Crystal violet staining of cells treated with various concentrations of 6c for 6 h. (**F**) Cells were treated with 6c (10 μM) for 6 h, then 6c was removed. Cells were subsequently culture for 24 h. Images were captured.

To visualize the effect of 85 derivatives of naphthalimides (10 μM), which are known to interact with DNA, obtained from our compound library on cancer cells, HepG2 cells were stained with acridine orange (AO), a nucleic acid-selective fluorescent dye (~488 nm) which emits green fluorescence when in monomer form. Cells were examined via HCS analysis instrument platforms after treatment for 6 h. Interestingly, as shown in Figure 1A, we found that the diffusion of green fluorescence occurred throughout the cytoplasm in cells exposed to 6c, the chemical structure of which was shown in Figure 1B, compared with the untreated cells. To our surprise, we also found that there was no green fluorescence in some part of the cell exposed to 6c. To define this observation, transmission electron microscopy (TEM) was used. Vacuolations with different sizes were observed in 6c-treated cells (Figure 1C). Further investigations showed that intracellular vacuolations were observed after exposure to 6c (10 μM) for 4 h in HepG2 cells; vacuolation size and numbers increased in a time-dependent manner (Figure 1D). Crystal violet staining displayed that 6c even at 5 μM induced the formation of vacuolations. Cytoplasmic displacement and eventually cell rupture occurred after cells were exposed to 6c at 20 μM (Figure 1E). To determine whether the vacuolations disappeared after withdrawing 6c, HepG2 cells were treated with 6c (10 μM) for 6 h, washed with PBS, and subsequently cultured for 24 h in medium without 6c. Extensive cytoplasmic vacuolations were still observed (Figure 1F).

### 2.2. Compound 6c Induced the Formation of Autophagosome

**Figure 2:**
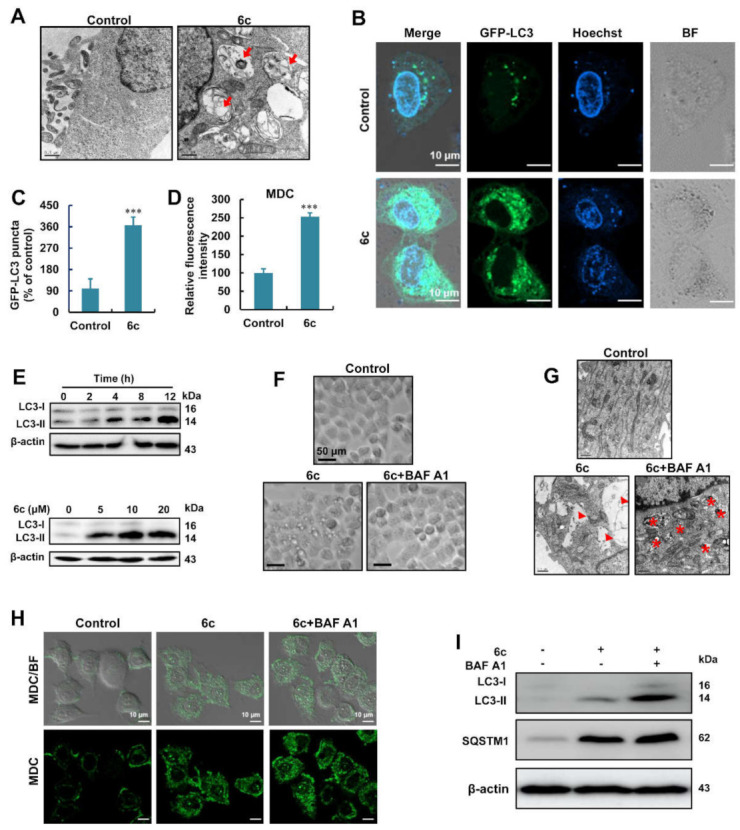
Compound 6c induced autophagosome formation. (**A**) TEM images of HepG2 cells treated with or without 6c for 6 h. (**B**,**C**) Images and statistical results of GFP-LC3 puncta in HepG2 cells treated with 6c (*n* = 25). (**D**) Statistical results of MDC staining in 6c-treated HepG2 cells. Cells exposed to 6c for 6 h were stained with MDC and then detected using HCS (*n* = 3). (**E**) Expression of LC3 in HepG2 cells treated with 6c at different time (up) or treated with various concentrations of 6c (down) for 24 h. (**F**) Pre-treatment with BAF A1 reduced the cytoplasmic vacuolations in HepG2 cells exposed to 6c for 6 h. (**G**) TEM images showed that BAF A1 (50 nM) pre-treatment for 1 h delayed the occurrences of cytoplasmic vacuolations induced by 6c for 6 h. (**H**) Images of MDC staining in BAF A1-treated HepG2 cells. Cells were exposed to BAF A1 for 1 h followed by 6c (20 μM) treatment for 6 h. (**I**) Expression of LC3 and SQSTM1 in HepG2 cells. Cells were exposed to BAF A1 for 1 h followed by 6c (20 μM) treatment for 24 h. *** *p* < 0.001 versus control.

Cytoplasmic vacuolations are derived from a variety of cellular organelles upon exposure to pharmaceutical agents and other chemicals [18]. TEM images showed that 6c-induced vacuolations are clear and contain visible cytoplasmic proteins and/or organelle components in their structures, thus suggesting the presence of autophagic vacuolations (Figure 2A). Autophagosomes can be determined by examining membrane-bound LC3-phospholipid conjugates. The number of green fluorescent protein (GFP)-LC3 puncta in HepG2 cells transfected with GFP-LC3 plasmids increased after treatment with 6c (Figure 2B,C). Compound 6c-induced autophagosome formation was further confirmed using MDC (Figure 2D), a fluorescent dye used as a marker for autophagy that specifically stains autophagosomes. Treatment with 6c also yielded a time- and concentration-dependent increase in the expression of LC3-II, which is the processed form of LC3 (Figure 2E). Next, we examined the effect of BAF A1, a late-stage inhibitor of autophagic flux, on autophagy in 6c-treated cells. We found that pre-treatment with BAF A1 eliminated the formation of 6c-induced cytoplasmic vacuolations (Figure 2F), which was confirmed via TEM images (Figure 2G). In addition, we found that BAF A1 pre-treatment elevated 6c-induced autophagosomes in HepG2 cells (Figure 2G), which was further evidenced by the increased fluorescence intensity of MDC in 6c-treated cells after BAF A1 pre-treatment (Figure 2H). Furthermore, BAF A1 pre-treatment resulted in the increase in 6c-induced LC3-II and sequestosome-1 (SQSTM1) in HepG2 cells (Figure 2I). These results suggested that 6c induced the formation of autophagosomes, but no autophagy degradation was found in 6c-treated HepG2 cells.

### 2.3. Autophagy Served as A Key Regulator in 6c-Induced Cell Death

**Figure 3:**
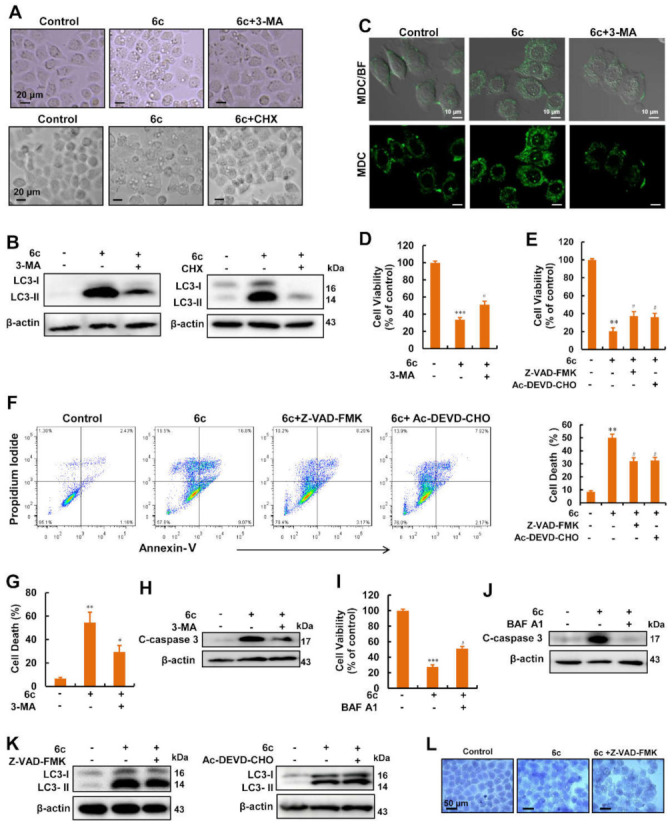
Autophagy was responsible for 6c-induced cell death. (**A**) Effects of 3-MA and CHX on 6c-induced cytoplasmic vacuolations, HepG2 cells were pre-treated with 3-MA (5 mM) or CHX (40 μM) for 1 h before 6c addition. (**B**) Expression of LC3 in HepG2 cells treated with 6c after pre-treatment with 3-MA (5 mM) or CHX (40 μM) for 1 h, respectively. (**C**) Images of MDC staining in HepG2 cells. Cells were pre-treated with 3-MA (5 mM) for 1 h before 6c (20 μM) treatment. (**D**) 3-MA attenuated 6c-induced cell viability inhibition (*n* = 3). Cells were pre-treated with 3-MA (5 mM) for 1 h followed by treatment with 6c (20 μM) for 24 h. Cell viability was examined using MTT assays. (**E**) Caspase inhibitors reduced the inhibitory effect of 6c on cell viability to a certain degree. After pre-treatment with caspase inhibitors Z-VAD-FMK (10 μM) and Ac-DEVD-CHO (10 μM) for 1 h, cells were exposed to 6c (20 μM) for 24 h. Cell viability was examined via MTT assays. (**F**) Caspase inhibitors Z-VAD-FMK and Ac-DEVD-CHO reduced cell death induced by 6c. After pre-treatment with caspase inhibitors Z-VAD-FMK (10 μM) and Ac-DEVD-CHO (10 μM) for 1 h, cells were exposed to 6c (20 μM) for 24 h. Cells were stained with Annexin V and PI, and analysis was performed using flow cytometry. (**G**,**H**) 3-MA attenuated 6c-induced cell death. Cell death was examined by Annexin V/PI staining and Western blot, respectively. (**I**,**J**) BAF A1 reduced 6c-induced cell viability inhibition and cleaved caspase 3 expression. Cells were pre-treated with BAF A1 (50 nM) for 1 h followed by treatment with 6c (20 μM) for 24 h. Cell viability was tested using MTT assays, the expression of cleaved caspase 3 was detected using Western blot. (**K**) Effects of Z-VAD-FMK and Ac-DEVD-CHO on 6c-induced LC3 expression. Cells were pre-treated with Z-VAD-FMK (10 μM) or Ac-DEVD-CHO (10 μM) for 1 h followed by treatment with 6c (20 μM) for 24 h. (**L**) Effects of Z-VAD-FMK on 6c-induced cytoplasmic vacuolations in HepG2 cells. Cells were pre-treated with Z-VAD-FMK (10 μM) for 1 h followed by treatment with 6c (20 μM) for 24 h. ** *p* < 0.01 and *** *p* < 0.001 vs. untreated groups; ^#^ *p* < 0.05 vs. 6c treatment alone.

We used 3-MA and cycloheximide (CHX), a protein biosynthesis inhibitor that inhibits the early stages of autophagy [19] to treat cells before 6c addition, and we found that pre-treatment with 3-MA and CHX for 1 h reduced the presence of 6c-induced vacuolations (Figure 3A). We also found that pre-treatment with 3-MA and CHX reversed the increase in 6c-induced LC3-II expression (Figure 3B). Meanwhile, pre-treatment with 3-MA reduced the fluorescence intensity of MDC induced by 6c (Figure 3C).

To determine the role of autophagy in 6c-induced cell death, 3-MA was added before 6c treatment; the results of cell viability assays obtained with the plate reader displayed that pre-treatment with 3-MA partially reversed the reduction in cell viability induced by 6c (Figure 3D). Our previous study reported that 6c induced the expression of cleaved caspase 3 [17]; to investigate whether caspase activation is an indispensable factor for 6c-induced apoptosis, Z-VAD-FMK, a caspase pan-inhibitor, and Ac-DEVD-CHO, a caspase 3 inhibitor, were used before treating with 6c. As shown in Figure 3E, pre-treatment with Z-VAD-FMK and Ac-DEVD-CHO partially attenuated the inhibitory effects of 6c on cell viability, respectively. Moreover, the number of dead cells was reduced after pre-treatment with Z-VAD-FMK and Ac-DEVD-CHO (Figure 3F). These results suggested that activation of caspase is involved in 6c-induced cell death. Notably, pre-treatment with 3-MA and BAF A1 reduced 6c-induced cell death obtained via flow cytometry, which was consistent with the results of the reduced expression of cleaved caspase 3 induced by 6c (Figure 3G–J). Interactions between different autophagy- and apoptosis-related proteins or genes and the corresponding signaling pathways have been identified, implying crosstalk occurs between these two cellular processes [20]. To test the role of apoptosis in 6c-induced autophagy, we treated cells with Z-VAD-FMK or Ac-DEVD-CHO before adding 6c. As shown in Figure 3K,L, pre-treatment with caspase inhibitors failed to attenuate 6c-induced the increase in LC3-II expression and cytoplasmic vacuolations. Taken together, these results emphasized the importance of stimulating autophagy during cell death.

### 2.4. Compound 6c Induced DNA Damage in Autophagy-Independent Manner

**Figure 4:**
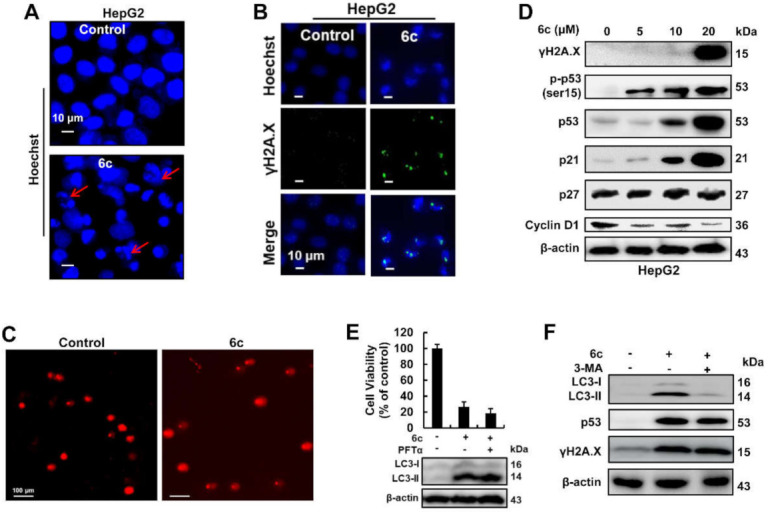
Compound 6c induced DNA damage in HepG2 cells. (**A**) Shape changes of nucleus after 6c (20 μM) treatment for 24 h in HepG2 cells. 6c-treated cells were stained with Hoechst 33342, and the images were captured using confocal microscopy. (**B**) The foci of γH2A.X in HepG2 cells treated with or without 6c (20 μM) for 24 h were detected using confocal microscopy. (**C**) Representative images of alkaline comet assay. HepG2 cells were treated with or without 6c (20 μM) for 24 h. (**D**) The expression of proteins related with DNA damage and p53 pathway after 6c treatment for 24 h was determined using Western blot. (**E**) Effects of p53 inhibitor PFTα on 6c-induced cell viability inhibition (up) and LC3-II expression (down). HepG2 cells were pretreated with PFTα (10 μM) for 1 h followed by treatment with 6c (20 μM) for 24 h. Cell viability was tested using MTT assays, and the expression of LC3 was detected using Western blot. (**F**) The expression of LC3, p53, and γH2A.X was determined using Western blot. HepG2 cells were pretreated with 3-MA for 1 h followed by treatment with 6c (20 μM) for 24 h.

DNA is vulnerable to damage resulting from many factors, including chemical agents and oxidative stress, and is involved in apoptosis, necrosis, and autophagy [9,21]. Treatment with 6c (20 μM) triggered nuclei shrinking and disintegration, as well as DNA internucleosomal fragmentation, as shown via Hoechst 33342 staining (Figure 4A). In 6c-treated cells, the fluorescence intensity associated with the phosphorylated histone variant H2AX on ser-139 (γH2A.X) was stronger than that in untreated cells (Figure 4B), indicating double-stranded DNA damage, which was further confirmed by comet assay (Figure 4C). The same pattern of γH2A.X expression was also observed in 6c-treated cells, as shown via Western blot (Figure 4D). Meanwhile, 6c treatment resulted in phosphorylation of ser 15 on p53 (Figure 4D), representing an early post-translational modification of p53 after DNA damage [22]. These data suggested that treatment with 6c caused DNA damage. As an important transcriptional factor, p53 could directly activate the transcription of many genes, and it also could attenuate the expression of many genes via activation of its target microRNAs [23,24,25]. In accordance with this, 6c treatment differentially regulated the protein expression of p21 and Cyclin D1, downstream targets of p53, following the increase in p53 expression that has been reported in our previous study [17]. Co-treatment with PFTα, a p53 inhibitor, and 6c did not affect the inhibition of cell viability and the expression of LC3-II induced by 6c (Figure 4E). Additionally, pre-treatment with 3-MA reversed the expression of LC3-II, but not p53 and γH2A.X induced by 6c (Figure 4F).

### 2.5. ROS Played an Important Role in 6c-Mediated Protein Expression

**Figure 5:**
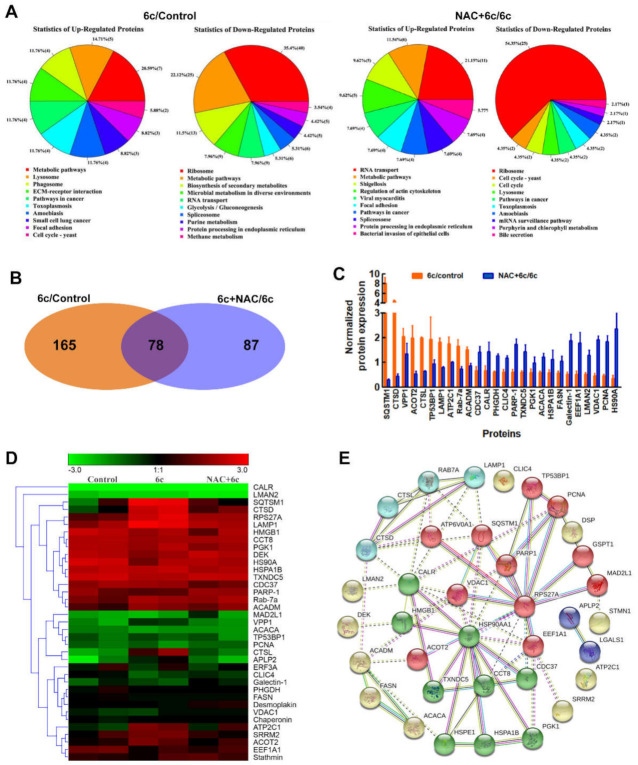
ROS mediated the expression of proteins in HepG2 cells treated with 6c (20 μM) for 24 h. In order to explore the role of ROS in 6c-treated HepG2 cells, cells were pretreated with NAC (10 mM) for 1 h before 6c addition. (**A**) Statistics of KEGG pathways of up-regulated and down-regulated differential expressed proteins in 6c/control group and NAC+6c/6c groups, respectively. (**B**) Number of differentially expressed proteins and the set relation. (**C**) The relative expression of selective proteins in 6c/control group and NAC+6c/6c group. (**D**) Hierarchical clustering analysis of selective protein changes in observations. (**E**) Protein–protein interaction networks were analyzed from the STRING database.

To further elucidate the underlying mechanisms associated with the effects of 6c, we used iTRAQ-based comparative proteomics, which has proven value in discovery-based proteomics. We previously found that 6c treatment increased the levels of ROS [17], in order to explore the precise function of ROS in 6c-treated cells, cells were pre-treated with NAC before 6c addition. We quantitatively identified a total of 4721 and 4720 proteins in the 6c/control and NAC+6c/6c groups, respectively. Among them, 3901 proteins contained at least two unique peptides. There were 243 differentially expressed proteins in the 6c/control group, and 165 differentially expressed proteins in the NAC+6c/6c group. All the identified proteins were subjected to bioinformatic analysis using the Gene Ontology (GO) database, the Kyoto Encyclopedia of Genes and Genomes (KEGG) database, and the Cluster of Orthologous Groups of proteins (COG) database. Function enrichment analysis (KEGG enrichment) of these differentially expressed proteins showed enrichment in several biological pathways, characterizing the most significant functional roles and related pathways. Statistics associated with up- and down-regulated differentially expressed proteins found in the KEGG pathways from the 6c/control and NAC+6c/6c groups were shown in Figure 5A. We compared the relationship between differentially expressed proteins and found that 78 proteins were expressed in both the 6c/control and NAC+6c/6c groups, including 56 proteins altered by NAC pre-treatment (Figure 5B). Proteins altered in the NAC pre-treatment group accounted for nearly 23% of those in the group treated with 6c alone and 34% of those in the NAC+6c/6c group. SQSTM1, a differentially expressed protein showing the most significant changes after treatment with 6c or pre-treatment with NAC (Figure 5C), is involved in autophagic cell death [26]. The proteins involved in mitochondrial function, autophagy, apoptosis, and proliferation were analyzed via hierarchical clustering (Figure 5D), including Ras-related protein Rab 7A, cathepsin D (CTSD), voltage-dependent anion-selective channel protein 1 (VDAC1), PARP-1, proliferating cell nuclear antigen (PCNA), Galectin-1, and Elongation factor 1-alpha (EEF1A1), which are important factors in cancer progression [27,28,29,30,31]. We further analyzed protein–protein interaction networks using data from the STRING database (Figure 5E). Taken together, these results confirmed that the ROS were critical mediators in 6c-treated cancer cells.

### 2.6. Compound 6c Induced Mitochondrial and Lysosomal Dysfunction Depending on ROS

**Figure 6:**
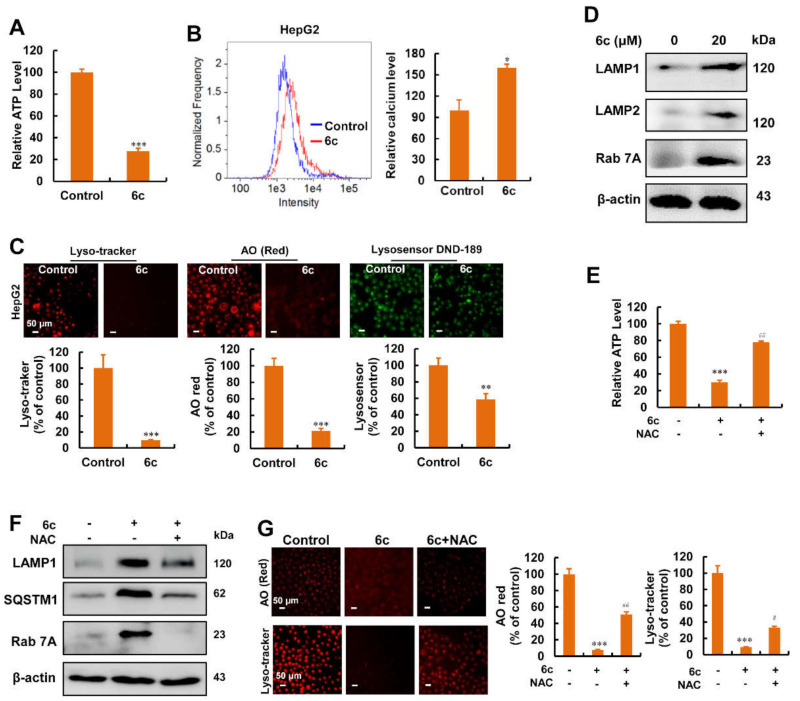
Compound 6c induced mitochondrial and lysosomal dysfunctions attributing to ROS overproduction. (**A**) 6c treatment resulted in ATP depletion. After treatment of 6c (20 μM) for 6 h, HepG2 cells were collected and mixed with cell titer Glo reagents. Luminescence data were recorded by using monochromator microplate reader (*n* = 3). (**B**) Flow cytometry analysis of mitochondrial calcium levels examined using Rhod 2 staining. Cells treated with 6c for 6 h were stained with Rhod 2 (*n* = 3). (**C**) Statistical intensity of lysosome tracker, AO, and lysosensor DND-189 in 6c-treated cells. HepG2 cells were treated with 6c (20 μM) for 6 h, the fluorescence intensity was detected using HCS (*n* = 3). (**D**) The expression of proteins related to lysosomes was examined using Western blot. HepG2 cells were treated with 6c (20 μM) for 24 h. (**E**) NAC pre-treatment increased ATP levels in 6c-treated cells. Cells were incubated with NAC (10 mM) for 1 h before 6c (20 μM) treatment for another 6 h (*n* = 3). (**F**) The expression of LAMP1, SQSTM1, and Rab 7A was determined using Western blot. HepG2 cells were pre-treated with NAC for 1 h followed by treatment with 6c (20 μM) for 24 h. (**G**) NAC (10 mM) partially improved the decreased fluorescence intensity of AO and lyso-tracker red induced by 6c (*n* = 3). * *p* < 0.01, ** *p* < 0.01 and *** *p* < 0.01 vs. control; ^#^ *p* < 0.05 and ^##^ *p* < 0.01 vs. 6c treatment alone.

iTRAQ-based quantitative proteomics revealed that 6c regulated the pathways which are related to metabolic pathways and lysosomes. Mitochondria are classically recognized as bioenergetic and biosynthetic organelles; thus, we postulated that 6c might regulate the function of mitochondria and lysosomes. In this study, we found that treatment with 6c for 6 h resulted in the depletion of ATP (Figure 6A). Mitochondria are also involved in calcium homeostasis, and an overload of calcium triggers mitochondria dysfunction. Next, we found that treatment with 6c increased the levels of calcium in mitochondria (Figure 6B). By tracking lysosomes with lysotracker red, a fluorescent dye that stains acidic organelles in living cells [32], we found that 6c treatment reduced lysotracker red puncta (Figure 6C). Similar results were obtained using AO (Figure 6C), a lysosomal tropic dye [33]. We further examined lysosomal pH using lysosensor DND-189, which emits a green fluorescence that intensifies under acidic conditions [34]. The fluorescent intensity of DND-189 was lower in 6c-treated cells than that in untreated cells (Figure 6C). Consistent with the results of iTRAQ-based quantitative proteomics, we found that 6c elevated the expression of lysosomal associated membrane protein (LAMP)1, LAMP2, and Rab 7A (Figure 6D). Moreover, the depletion of ATP, the altered expression of LAMP1, SQSTM1, and Rab 7A, as well as the decrease in fluorescence intensity of AO and lyso-tracker in 6c-treated cells were reversed by NAC pre-treatment (Figure 6E–G). Taken together, these results demonstrated that 6c induced mitochondrial and lysosomal dysfunction depending on ROS.

### 2.7. Compound 6c-Induced Vacuolations, Autophagosome Formation, Cell Death, and DNA Damage Were Reversed by Antioxidants

**Figure 7:**
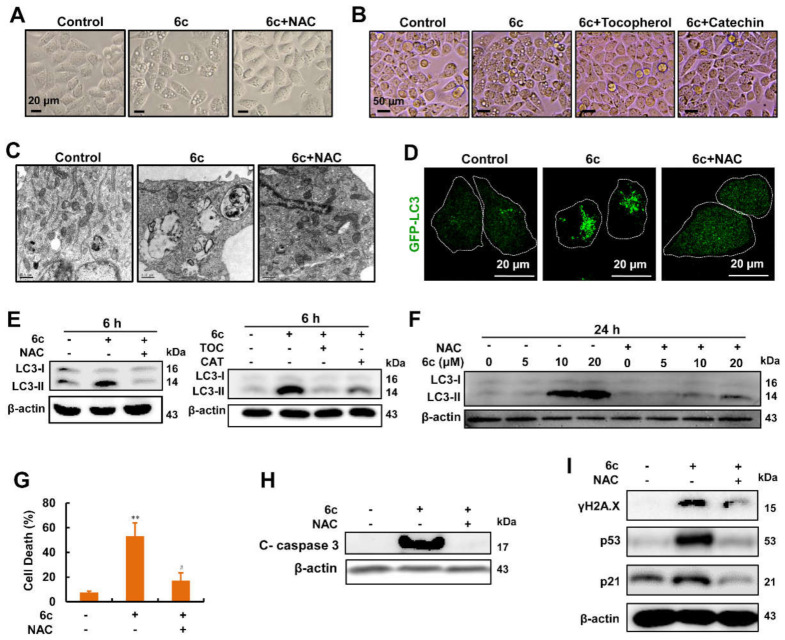
Compound 6c exerted its functions depending on ROS. (**A**) NAC abolished the cytoplasmic vacuolations induced by 6c in HepG2 cells. Cells were pre-treated with NAC (10 mM) for 1 h followed by 6c (20 μM) treatment for 6 h. (**B**) α-tocopherol (TOC) and catechin (CAT) abolished the cytoplasmic vacuolations induced by 6c in HepG2 cells. Cells were pre-treated with TOC and CAT for 1 h followed by 6c (20 μM) treatment for another 6 h. (**C**) TEM images showed that NAC (10 mM) reduced the formation of vacuolations and autophagosome induced by 6c (20 μM). (**D**) NAC weakened 6c-induced GFP-LC3 puncta in HepG2 cells. Transfected HepG2 cells were pre-treated with NAC (10 mM) for 1 h followed by 6c (20 μM) treatment, and images of GFP-LC3 puncta were captured using confocal microscopy. (**E**) NAC, TOC, and CAT decreased the increased expression of LC3-II in HepG2 cells treated with 6c (20 μM) for 6 h. (**F**) NAC reversed the increased expression of LC3-II in HepG2 cells treated with 6c for 24 h. (**G**,**H**) 6c-induced cell death was weakened by NAC pre-treatment. Cells were pre-treated with NAC (10 mM) for 1 h followed by 6c (20 μM) treatment for another 24 h, and cell death was determined by staining Annexin V/PI using flow cytometry (*n* = 3). The expression of cleaved caspase 3 was detected using Western blot. (**I**) NAC diminished the increased expression of γH2A.X, p53, and p21 induced by 6c. After pre-treatment with NAC (10 mM) for 1 h, cells were further treated with 6c (20 μM) for 24 h. ** *p* < 0.01 vs. control; ^#^ *p* < 0.05 vs. 6c treatment alone.

In this study, we found that pretreatment with antioxidants, including NAC, α-tocopherol, and catechin, blocked 6c-induced cytoplasmic vacuolations (Figure 7A,B). ROS can stimulate autophagy [35] and mediate cell death [36], implying that ROS play an important role in cellular fate. TEM images of cells pre-treated with NAC not only confirmed the reduction in vacuolations, but also displayed the decrease in autophagosome formation (Figure 7C). In GFP-LC3-transfected HepG2 cells, fewer GFP-LC3 puncta were observed in cells treated with 6c and NAC than that in cells treated with 6c alone (Figure 7D), which is consistent with the above results. Western blot analysis showed that LC3-II expression was repressed in the presence of antioxidants in HepG2 cells exposed to 6c (Figure 7E,F). Moreover, we found that NAC reduced cell death induced by 6c, which was further verified by the expression of cleaved caspase 3 (Figure 7G,H). We also found that NAC abated the ability of 6c treatment to increase the expression of γH2A.X, p53, and p21 (Figure 7I). The expression of cleaved caspase 3 and p53 in the NAC pre-treatment system is consistent with our previous observations [17]. These results demonstrated that 6c exerted its function depending on ROS.

### 2.8. Mitochondria Were the Main Source of ROS Generation Induced by Compound 6c

**Figure 8:**
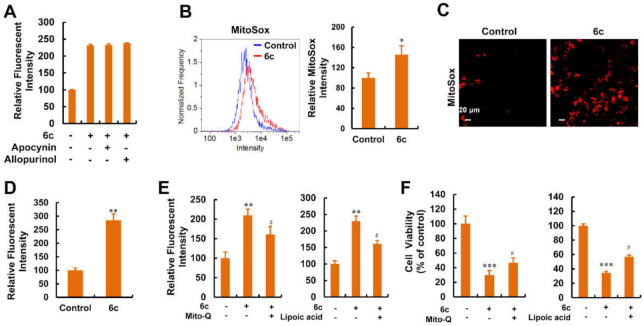
Compound 6c induced mitochondrial ROS generation. (**A**) HepG2 cells were pre-treated by apocynin (300 μM) and allopurinol (20 μM) for 1 h before 6c addition. After treatment with 6c (20 μM) for 6 h, cells were stained with DCFH-DA and detected using HCS. (**B**) HepG2 cells were treated with 6c (20 μM) for 6 h, the mitochondrial ROS was detected by MitoSox staining using flow cytometry (*n* = 3). (**C**,**D**) HepG2 cells were treated with 6c (20 μM) for 6 h, the mitochondrial ROS was detected by MitoSox staining using HCS (*n* = 3). (**E**) Effects of antioxidants on 6c-induced ROS levels. Before treatment of 6c (20 μM) for 6 h, HepG2 cells were pre-treated with Mito-Q (1 μM) and alpha-lipoic acid (200 μM) for 1 h, respectively. Cells were stained with DCFH-DA and the fluorescence intensity was detected using HCS (*n* = 3). (**F**) Antioxidants reversed 6c-induced cell viability inhibition to varying degrees. After pre-treatment of Mito-Q (1 μM) and alpha-lipoic acid (200 μM) for 1 h, respectively, HepG2 cells were exposed to 6c (20 μM) for another 24 h followed by the detection of cell viability using MTT assays (*n* = 3). * *p* < 0.05, ** *p* < 0.01 and *** *p* < 0.001 vs. untreated groups; ^#^ *p* < 0.05 vs. 6c treatment alone.

The source of 6c-induced ROS in HepG2 cells is unknown. As shown in Figure 8A, pre-treating cells with apocynin, a NADPH oxidase inhibitor, and allopurinol, a xanthine oxidase inhibitor, did not affect 6c-induced ROS generation [37]. Administered 6c localized within the mitochondria, which are major contributors of endogenous ROS in cancer cells [17,38]. Therefore, we postulated that the increase in ROS generation originated from the mitochondria. To confirm this conjecture, we used MitoSox fluorescent intensity as an indicator for mitochondrial ROS in live cells [39] via flow cytometry and HCS. As expected, 6c significantly enhanced the MitoSox fluorescent intensity in HepG2 cells (Figure 8B–D). Additionally, mitoquinone (Mito-Q), a mitochondria-selective ROS scavenger, reduced intracellular ROS (Figure 8E). Alpha-lipoic acid, which is found naturally in the mitochondria and is an essential cofactor for mitochondrial enzymes, serving as an anti-oxidant by participating in ROS scavenging [40,41]. Like Mito-Q, alpha-lipoic acid reduced 6c-induced ROS (Figure 8E). More importantly, these antioxidants attenuated the inhibitory effect of 6c on cell viability to varying degrees (Figure 8F).

## 3. Discussion

**Figure 9:**
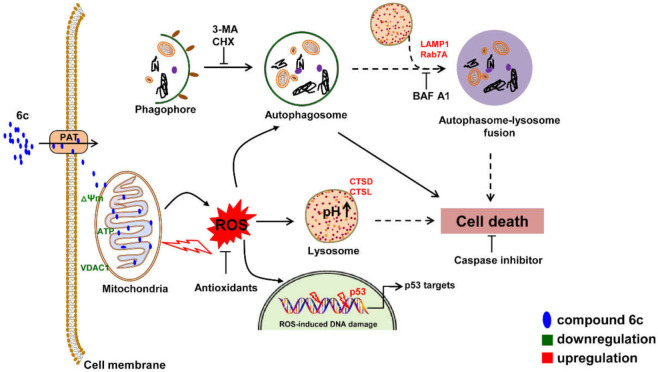
Schematic model for 6c in regulation of ROS, mitochondrial and lysosomal function, autophagy, cell death, and DNA damage.

In this study, we displayed that 6c induced mitochondrial ROS generation, which in turn caused mitochondrial and lysosomal dysfunction. 6c induced the formation of autophagosomes, resulting in cell death. 6c also stimulated DNA damage. Our study demonstrated the pivotal role of mitochondrial ROS in the organelle homeostasis, autophagy, cell death, and DNA damage. Schematic models for 6c in regulation of ROS, mitochondrial and lysosomal function, autophagy, cell death, and DNA damage were shown in Figure 9.

Autophagy defects in cancers are associated with malignant differentiation and a poor prognosis [42,43]. During stress conditions, autophagy is a tightly regulated pathway that maintains cellular homeostasis via lysosomal degradation of damaged cellular organelles and long-lived proteins [44]. In HepG2 cells, we found that treatment with 6c induced the expression of LC3-II, the formation of GFP-LC3 puncta and the formation of autophagosomes, suggesting that 6c induced the initiation of autophagy. In the late stage of autophagy, the autophagosome fuses with the lysosome to form an autolysosome, which is the main proteolytic system. Suppression of this process impairs autophagic degradation [45]. Rab 7A plays an important role in autophagosome–lysosome fusion [30,46]. Although treating cells with compound 6c increased Rab 7A expression, compound 6c induced the expression of SQSTM1, which is always used to monitor autophagic flux, implying that 6c might induce inefficient autophagy. Therefore, exploring the underlying mechanisms of anticancer drug-mediated autophagy will provide a novel therapeutic strategy for cancer treatment via autophagy.

Intra-lysosomal pH is an important factor for maintaining lysosomal function, and it is generally considered that the pH of acidic compartments is critical for autophagosome–lysosome fusion [47,48]. However, the recent study displayed that lysosomal acidification is not a prerequisite for fusion [49]. In a manner similar to IMB-6G and liensinine [50,51], compound 6c increased the expression of CTSD and CTSL, which are the important proteases in lysosomes. The changes in lysosomal pH and cathepsin expression illustrated that the lysosomal functions were altered in the 6c-treated system. A recent paper reported that lysosomes are dysfunctional with the decreased expression of LAMP1 and LAMP2 [52]. Interestingly, the expression of LAMP1 and LAMP2 was increased after 6c treatment; however, the fluorescence intensity of AO (red), lyso-tracker, and DND-189 was reduced. Xiu-Tang Cheng et al. demonstrated that LAMP1 intensity, trafficking, and distribution do not necessarily represent degradative lysosomes or autolysosomes under pathological conditions [53]. Thus, in 6c-treated HepG2 cells, how the function of lysosomes is regulated is worthy of in-depth study.

Naphthalimides are known as potential DNA interactive agents involved in DNA repair and DNA damage [54]. In this study, for the first time, we demonstrated that compound 6c at high concentrations induced DNA damage, as verified by comet assays, the increased foci and expression of γH2A.X, as well as the augmentation of phospho-p53 (ser15) in 6c-treated cells. The increased expression of phospho-p53 (ser15) might result from the increase in total p53 expression, which was reported in our previous study [17]. Moreover, NAC treatment attenuated the increased expression of 6c-induced γH2A.X, confirming that DNA is an important target of elevated ROS [55]. Many reports have shown that DNA damage induces autophagy; however, the exact role of autophagy in DNA damage is unknown [56]. Inactivation of p53 using the specific inhibitors did not affect cell proliferation inhibition and the expression of LC3-II in 6c-treated cells. However, the expression of p53 induced by 6c was slightly reversed by treatment with 3-MA. Moreover, 6c-induced γH2A.X was not altered in 3-MA treated system. Surprisingly, 3-MA pre-treatment reduced the presence of cytoplasmic vacuolations, enhanced cell viability, and reduced cell death in 6c-treated cells, suggesting that 6c induced cell death through autophagy. p53 also plays an important role in senescence [57]. Thus, these results inspired us to further explore the complicated relationship between DNA damage, senescence, autophagy, and cell death in 6c-treated HepG2 cells.

Elevated cellular ROS levels can be derived from mitochondria, NADPH oxidases, cyclooxygenases, xanthine oxidases, lipoxygenases, and cytochrome P450 enzymes [58]. In our study, Mito-Q and alpha-lipoic acid, but not apocynin and allopurinol, attenuated 6c-induced ROS generation, implying that the mitochondria served as the main source of ROS, which supported our previous hypothesis [17]. Induction of mitochondrial ROS production by compound 6c was confirmed using MitoSox, a specific probe for mitochondrial ROS. Therefore, compound 6c might target mitochondria complex I to V or mitochondrial components in HepG2 cells, resulting in the generation of ROS. Although the data suggested that the ROS were derived from mitochondria, further experiments should be carried out to investigate the targeted proteins or pathways by which compound 6c enhanced mitochondrial ROS generation.

Excess ROS can have detrimental consequences, including mitochondrial damage and cell injury [59]. Elevated ROS in 6c-treated cells resulted in ATP depletion and ΔΨm disruption [17], as well as alkalinization of lysosomal pH. These results showed the homeostasis of mitochondria and lysosomes were disturbed by compound 6c via ROS. Importantly, ROS scavengers reduced 6c-induced vacuolations, accumulation of LC3-II, cell death, and DNA damage. Therefore, ROS are the important regulators in the function of compound 6c.

## 4. Materials and Methods

### 4.1. Cell Lines, Cell Culture and Reagents

HepG2 cells were purchased from the Cell Bank of the Chinese Academy of Science (Shanghai, China) and cultured in RPMI-1640 medium. All media were supplemented with 10% FBS, 100 units/mL penicillin and 100 units/mL streptomycin (Solarbio, Beijing, China). FBS was obtained from GIBCO (Grand Island, NY, USA). In this study, cells were pretreated with inhibitors, including BAF A1 (50 nM), NAC (10 mM), Mito-Q (1 μM), alpha-lipoic acid (200 μM), apocynin (300 μM), allopurinol (20 μM), α-tocopherol (TOC, 5 mM), catechin (CAT, 50 μM), 3-MA (5 mM), cycloheximide (CHX, 40 μM), and PFTα (10 μM), for 1 h followed by treatment with 6c.

### 4.2. ATP Content Detection

The cell titer Glo™ (Promega, Madison, WI, USA) reagents were used to measure total ATP levels. Cells were plated into 12-well plates and treated with 6c at 20 μM. After treatment for 6 h, cells were collected and counted. Cells were mixed with cell titer Glo reagent (lysis buffer mixed with cell titer Glo substrate, diluted with PBS 1:1), and the mixture was transferred to 96-well black-walled plates. Luminescence data were recorded using a Clariostar monochromator microplate reader (BMG Labtech, Ortenberg, Germany).

### 4.3. GFP-LC3 Transfection

Cells were transfected with 2 μg of GFP-LC3-expressing plasmid in each well of a laser confocal culture dish using lipofectamine, according to the manufacturer’s instructions (Invitrogen). After 16 h, cells were treated with 6c, and the fluorescence of GFP or GFP-LC3 was viewed after 6c treatment for 8 h using a laser confocal microscopy (Leica, Heidelberg, Germany).

### 4.4. iTRAQ Quantification Analysis

After treatment with 6c (20 μM) for 24 h, protein samples were prepared. Total protein concentration was measured using the Bradford method. After protein digestion, equal volumes of 0.1% FA were added for acidization. Peptides were purified and dried with a vacuum concentration meter. Samples were labeled with iTRAQ Reagent-8 plex Multiplex Kit (AB Sciex U.K. Limited) according to the manufacturer’s instructions. The labeled samples were fractionated using a high-performance liquid chromatography system (Thermo DINOEX Ultimate 3000 BioRS) using a Durashell C18 (5 μm, 100 Å, 4.6 × 250 mm). Finally, 12 fractions were collected. LC-MS/MS Analysis and LC-ESI-MS/MS analysis were performed on an AB SCIEX nanoLC-MS/MS (Triple TOF 5600 plus) system. The original MS/MS file data were submitted to ProteinPilot Software v4.5 for data analysis. For protein identification, the Paragon algorithm 2, which was integrated into ProteinPilot, was employed against Uniprot Homo sapiens database for database searching. For biological replicates or technology replicates experiments, the average fold-change induced by treatment relative to the control was defined as -fold changes. Statistical significance of the difference in the levels of expression of proteins between samples to be compared was determined by Student’s t-test (two-tailed and unpaired); we took a 1.5-fold change and *p* value less than 0.05 as the threshold to identify significant changes. Hierarchical clustering was applied to differentially expressed proteins to determine the biological and functional properties of the identified proteins. To identify candidate biomarkers, we employed a hypergeometric test to perform KEGG pathway enrichment.

### 4.5. MDC Staining

Cells were incubated in the presence of 6c for 6 h and stained with the autofluorescent marker monodansylcadaverine (MDC) (Sigma Chemical Co., MO, USA) at a concentration of 50 μM in PBS at 37 °C for 40 min. MDC staining was observed using high content screening (HCS) or a laser confocal microscopy (Leica).

### 4.6. γH2A.X Staining

Cells were seeded into a cell culture dish, and after incubation for 24 h, 6c at the concentration of 0 μM and 20 μM was added. Cells were treated with 6c for 24 h, then fixed using 4% formaldehyde for 30 min. Fixed cells were washed three times with PBS and blocked in 0.5% bovine serum albumin. The primary specific antibody of γH2A.X was added, and cells and antibody were incubated at 4 °C overnight, followed by incubation with the secondary antibody. Then, Hoechst 33342 was added to the samples. Images were captured.

### 4.7. Annexin V-FITC/Propidium Iodide Staining

Cell apoptosis was evaluated using Annexin V-FITC/Propidium iodide (PI) staining detected via flow cytometry. Cells were seeded into 6-well plates at 1 × 10^5^ cells/well and treated for 24 h. Cells were harvested, centrifugated, and re-suspended with PBS. After centrifugation, cells were re-suspended with binding buffer and incubated with Annexin V and PI. Analysis was performed by flow cytometry (BD Biosciences, CA, USA).

### 4.8. Reactive Oxygen Species Detection

ROS was examined with fluorescent probe DCFH-DA (Sigma Chemical Co., MO, USA) or MitoSox (Thermo Fisher Scientific, CA, USA) detected by HCS, cells were seeded into 96-well plates at 1 × 10^4^ cells/well and were cultured for 24 h. 6c at a concentration of 20 μM was added. After treatment for 6 h, cells were washed three times with PBS, followed by staining with DCFH-DA or MitoSox in the dark for 15 min at room temperature. Then, cells were washed three times with PBS and were stained with Hoechst 33342. Stained cells were analyzed using HCS. The mitochondrial ROS level was also detected via flow cytometry using MitoSox staining.

### 4.9. Transmission Electron Microscope Assay

Treated HepG2 cells were fixed using 2.5% glutaraldehyde overnight at 4 °C and washed three times by cacodylate buffer. Then, cells were further fixed using 1% osmium tetroxide for 1 h at room temperature. Sections of 70 nm were cut on a Reichert Ultracut S Ultramicrotome (Leica, Heidelberg, Germany). The sections were stained with 4% uranyl acetate and Reynald’s lead citrate. Images were captured at 80 kV using a JEOL 1200 EX transmission electron microscope (Leica, Heidelberg, Germany).

### 4.10. Lysosomal Acidification Detection

Cells were seeded into 96-well plates at 1 × 10^4^ cells/well. After incubation for 24 h, cells were further treated for 6 h. Treated cells were stained with AO, lysosome-tracker red (Beyotime, Shanghai, China), and Lysosensor Green DND-189 (Yeasen Biotechnology Co. Ltd., Shanghai, China), and the fluorescence intensity were analyzed using HCS.

### 4.11. Western Blot

In brief, treated cells were harvested and centrifuged. Cell pellets were washed three times with ice-cold PBS and were lysed with RIPA buffer (Beyotime). The concentration of protein was examined using a BCA assay kit. The total lysates were subjected to 5×SDS-loading buffer at 100 °C for 10 min followed by exposure to 10–12% SDS-PAGE for 2 h. Proteins were transferred onto PVDF membranes, and membranes were blocked by 5% dried skimmed milk at room temperature for 1 h. After blockage, membranes were incubated with specific primary antibodies at 4 °C overnight. After incubation with determined HRP-conjugated secondary antibody, protein expression was detected by using the ECL plus reagents (Beyotime). Antibody against β-actin (sc-47778, 1:500) was purchased from Santa Cruz (CA, USA). Antibodies against p21 (10355-1-AP, 1:500), LC3 (14600-1-AP, 1:500), SQSTM1 (18420-1-AP, 1:1000), PARP-1 (13371-1-AP, 1:500), p53 (10442-1-AP, 1:500), Rab 7A (55469-1-AP, 1:1000), LAMP1 (21997-1-AP, 1:500), LAMP2 (66301-1-Ig, 1:500), Cyclin D1 (60186-1-Ig, 1:500), and p27 (25614-1-AP, 1:500) were purchased from Proteintech (Hubei, China). Anti-cleaved-caspase 3 (CY5031, 1:500) was purchased from Abways Technology (Shanghai, China). Antibodies against γH2A.X (AF1201, 1:1000) and p-p53 (AF5893, 1:500) were purchased from Beyotime.

### 4.12. Mitochondrial Calcium Detection

The levels of mitochondrial calcium were detected with fluorescent probe Rhod 2 (Thermo Fisher Scientific) using flow cytometry. Cells were seeded into 6-well plates and were cultured for 24 h. 6c at a concentration of 20 μM was added. After treated for 6 h, cells were stained with Rhod 2. Then, cells were washed three times with PBS and the fluorescence was analyzed using flow cytometry.

### 4.13. Comet Assay

The measurement of DNA damage was performed using the alkaline comet assay kit according to the instructions (KeyGen Biotech, NanJing, China). Briefly, HepG2 cells were treated with compound 6c or not for 24 h. Cells were washed and re-suspended using PBS to make a density of 1 × 10^6^ cells/mL. Cells were mixed with comet agarose and then loaded onto the slides followed by treatment with the lysis buffer and an alkaline solution. The damaged DNA was separated using alkaline gel electrophoresis. The slides were stained with PI, and the images were captured using fluorescence microscopy.

### 4.14. Data Analysis

All the data are presented as the mean ± SD and analyzed using by one-way ANOVA and Student’s *t*-test. *p* < 0.05 was considered to be significant statistically.

## 5. Conclusions

In summary, data from this study suggested an intricate relationship between mitochondria, lysosomes, ROS, autophagy, cell death, and DNA damage, all of which participate in the toxicity associated with compound 6c in cancer cells. Overproduction of mitochondrial ROS plays a critical role in these cellular responses. Our findings support the theory that the use of ROS-inducing agents such as compound 6c might be an appropriate strategy for cancer therapy.

## Data Availability

Not applicable.

## Abbreviations

ROS, reactive oxygen species; DCFH-DA, 2′,7′-dichlorodihydrofluorescein diacetate; NAC, N-acetyl-L-cysteine; 3-MA, 3-methyladenine; BAF A1, bafilomycin A1; ACD, autophagic cell death; PARP-1, poly [ADP-ribose] polymerase 1; mTOR, mammalian target of rapamycin; JNK, c-Jun N-terminal kinases; TOC, α-tocopherol; CAT, catechin; CHX, cycloheximide; MDC, monodansylcadaverine; GFP, green fluorescent protein; SQSTM1, sequestosome-1; KEGG, Kyoto Encyclopedia of Genes and Genomes; CTSD, cathepsin D; VDAC1, voltage-dependent anion-selective channel protein 1; PCNA, proliferating cell nuclear antigen; EEF1A1, Elongation factor 1-alpha; LAMP, lysosomal associated membrane protein; Mito-Q, mitoquinone.
